# Mutational and transcriptional profiling of cuproptosis-associated genes in amyotrophic lateral sclerosis

**DOI:** 10.1016/j.gendis.2024.101208

**Published:** 2024-01-14

**Authors:** Di He, Xilu Wang, Meng Hao, Dongchao Shen, Xunzhe Yang, Mingsheng Liu, Yi Li, Jiucun Wang, Liying Cui

**Affiliations:** Department of Neurology, Peking Union Medical College Hospital, Chinese Academy of Medical Sciences and Peking Union Medical College, Beijing 100730, China; State Key Laboratory of Genetic Engineering, Collaborative Innovation Center for Genetics and Development, School of Life Sciences and Human Phenome Institute, Fudan University, Shanghai 201203, China; Department of Neurology, Peking Union Medical College Hospital, Chinese Academy of Medical Sciences and Peking Union Medical College, Beijing 100730, China; State Key Laboratory of Genetic Engineering, Collaborative Innovation Center for Genetics and Development, School of Life Sciences and Human Phenome Institute, Fudan University, Shanghai 201203, China; State Key Laboratory of Genetic Engineering, Collaborative Innovation Center for Genetics and Development, School of Life Sciences and Human Phenome Institute, Fudan University, Shanghai 201203, China; Research Unit of Dissecting the Population Genetics and Developing New Technologies for Treatment and Prevention of Skin Phenotypes and Dermatological Diseases, Chinese Academy of Medical Sciences, Beijing 100730, China; Department of Neurology, Peking Union Medical College Hospital, Chinese Academy of Medical Sciences and Peking Union Medical College, Beijing 100730, China

Amyotrophic lateral sclerosis (ALS) is a neurodegenerative disorder characterized by progressive muscle weakness and atrophy.^1^ The widespread implementation of next-generation sequencing in clinical practice has facilitated the identification of over 40 ALS-associated genes, among which copper/zinc-superoxide dismutase 1 (*SOD1*) assumes a pivotal role in East Asian populations.^1^ Moreover, it is increasingly apparent that the intricate interplay between genetic predisposition and exposome over time may contribute significantly to the etiology of this debilitating disease. While the precise pathogenic role of copper (Cu) in ALS remains elusive, accumulating evidence suggests that Cu deficiency may be linked to *SOD1*-associated ALS, possibly mediated through the promotion of the hydrophobicity of mutant *SOD1*, ultimately exacerbating its neurotoxic aggregation.^2^ Furthermore, a novel form of regulated cell death, termed cuproptosis, has emerged as an intriguing phenomenon. Cuproptosis is characterized by the direct binding of Cu to the lipoylated enzymes of the tricarboxylic acid cycle, leading to mitochondrial lipoylated protein aggregation and consequent proteotoxic stress.^3^ Given that mitochondrial dysfunction constitutes a critical pathophysiological hallmark in ALS, the perturbation of Cu homeostasis may possibly play a prominent role in the context of *SOD1*-induced neurodegeneration.

To systematically investigate the potential links between Cu and ALS, we embarked on an analysis of the mutational landscape of genes associated with Cu homeostasis and cuproptosis. Our ALS cohort comprised 508 patients of Chinese descent, who were diagnosed between December 2017 and July 2021 at Peking Union Medical College Hospital based on the El Escorial criteria (Table S1). The patients were followed up until January 2023, and individuals displaying signs of other neurological disorders were carefully excluded from the study. The control cohort consisted of 4961 population-matched healthy individuals from the HuaBiao project.^4^ Whole-exome sequencing was conducted for all participants. A comprehensive cohort and methodology description can be accessed in the *Extended Materials*. Genes implicated in copper homeostasis were curated based on the Molecular Signature Database (MsigDB, v7.5) (GO: 0055,070 and WP3286, 59 genes in total), while the cuproptosis gene set encompassed 10 genes identified through the genome-wide CRISPR/Cas9 loss-of-function screening^3^ (Table S2). To assess the cumulative impact of rare variants within each gene, we employed a gene-based sequence kernel association test analysis. The results revealed that, after applying the Bonferroni correction, 44 out of 59 copper homeostasis genes and 9 out of 10 cuproptosis-associated genes displayed significant enrichment in rare variants (Table S3). This observation underscored a plausible connection between the pathogenesis of ALS and the biological pathways intricately linked to Cu metabolism, specifically emphasizing the role of cuproptosis in this context.

We then explored the correlation between genetic variations and clinical progression, with the variants satisfying the following criteria considered: (i) non-synonymous exonic variants annotated as missense, start-lost, stop-gained, stop-loss, or frameshift mutations; (ii) minor allele frequency lower than 0.001 in Genome Aggregation Database East Asian population and HuaBiao project; (iii) significant allelic association with ALS as determined by the standard Fisher's exact test. This selection process led to the identification of 141 rare Cu variants that met these criteria (Table S4). Excluding those specific to *SOD1*, 119 patients (23.4 %) bore rare variants within genes associated with Cu homeostasis or cuproptosis (Table S5). Notably, individuals carrying rare Cu variants exhibited a discernible trend toward a more rapid decline in ALS Functional Rating Scale scores at the time of diagnosis (Fig. 1A). This proclivity was further corroborated by survival analysis, which unveiled a noteworthy association between the presence of cuproptosis variants and unfavorable clinical outcomes (log-rank [Mantel–Cox] test, *P* = 0.0171) (Fig. 1B). After adjusting for covariates, the presence of rare cuproptosis variants remained as an independent predictor of mortality as determined through the Cox proportional hazards regression analysis (hazard ratio:1.88, 95 % confidence interval: 1.07 to 3.31), whereas no such association was observed in relation to Cu homeostasis variants (Fig. 1C).

**Figure 1:**
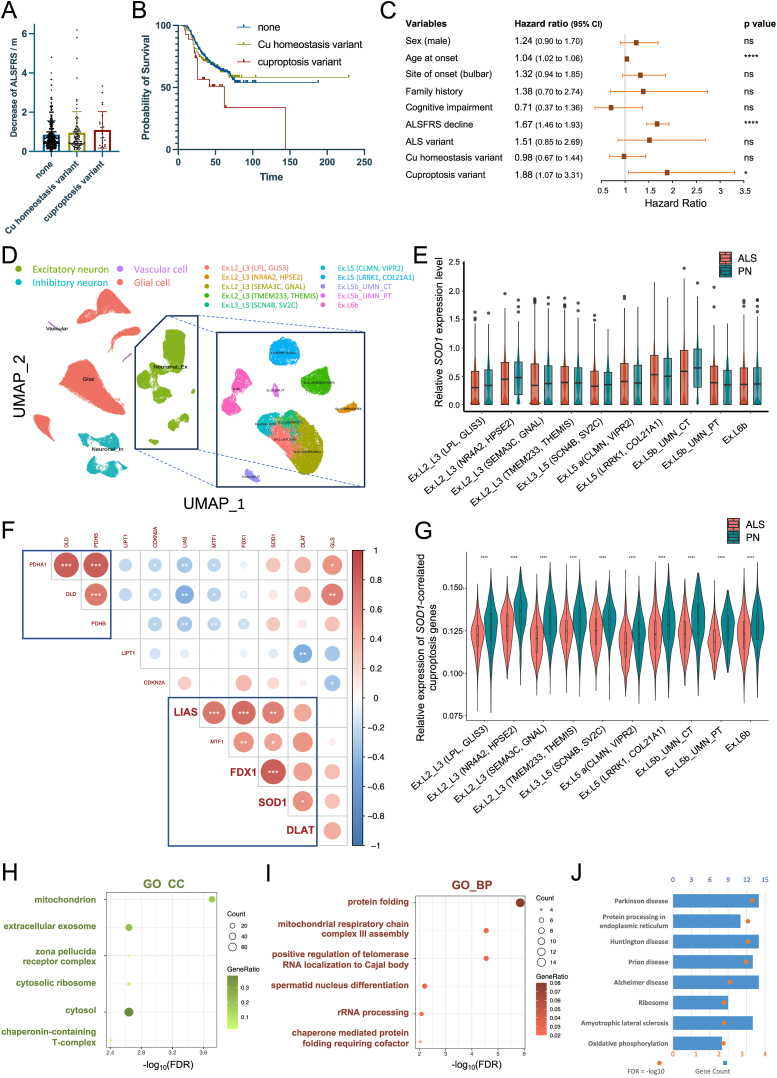
Clinical significance of Cu-related gene mutations and the expression of cuproptosis genes in the prefrontal motor cortex. **(A)** The bar plot comparing the rate of ALSFRS (ALS Functional Rating Scale) decline among patients carrying Cu variants. **(B)** Kaplan–Meier survival curves of ALS patients carrying Cu variants. **(C)** The forest plot representing the hazard ratio of the Cox proportional hazards model. The error bars were two-sided 95 % confidence intervals. The ALS variants were defined as disease-causing or likely disease-causing mutations (DM/DM?) according to the Human Gene Mutation Database (HGMD). **(D)** Uniform manifold approximation and projection (UMAP) plots of the identified cell populations in ALS and pathologically normal (PN) samples. **(E)** The boxplot comparing the *SOD1* expression between ALS and PN across excitatory neuron subtypes. **(F)** Correlation heatmap of expression between SOD1 and cuproptosis genes in excitatory neurons through Pearson correlation analysis. Red represents a positive correlation and blue a negative correlation. ^∗^*P* < 0.05, ^∗∗^*P* < 0.01, ^∗∗∗^*P* < 0.001. **(G)** The average expression of *FDX1*, *LIAS*, and *DLAT* in excitatory neurons calculated by AddModuleScore. **(H)** Dot plot of Gene Ontology (GO) cellular component (CC) enriched terms colored by *P* values. **(I)** Dot plot of GO biologic process (BP) enriched terms colored by *P* values. **(J)** Bar plot of Kyoto Encyclopedia of Genes and Genomes (KEGG) enriched terms colored by *P* values. ALS, amyotrophic lateral sclerosis.

To further substantiate the putative pathogenic roles of cuproptosis genes in ALS, we analyzed the single-cell RNA sequencing dataset (GSE174332) obtained from the primary motor cortex of both ALS-afflicted and pathogenically normal subjects.^5^ Following dimensional reduction and clustering predicated upon canonical cell markers of the central nervous system, cells were categorized into neurons (both excitatory and inhibitory), glial cells, and vascular cells. Considering the selective vulnerability of excitatory neurons in ALS pathophysiology, our focus narrowed to this specific cell population, further segregated into subclusters based on the cell markers delineated in the original study (Fig. 1D). No discernible difference in *SOD1* expression was observed between neurons derived from ALS-affected and pathogenically normal subjects, thereby suggesting that an excess or deficiency of wildtype *SOD1* in excitatory neurons is unlikely to be the primary pathogenic factor in ALS patients devoid of known *SOD1* mutations (Fig. 1E). We subsequently embarked on an exploration of the potential correlation between the expression of *SOD1* and cuproptosis genes within excitatory neurons. We observed a significant correlation between the expression of genes encoding the subunits of the pyruvate dehydrogenase complex (*PDHA1*, *PDHB*, and *DLD*), signifying a potential synergistic relationship in biological processes (Fig. 1F). Particularly noteworthy was a robust correlation observed between the expression of *SOD1* and *FDX1* (*r* = 0.84) and, to a lesser extent, with *LIAS* and *DLAT.*

Given their pivotal roles in mediating cuproptosis, coupled with their close correlation with *SOD1* in the motor cortex, we further evaluated the expression of *FDX1*, *LIAS*, and *DLAT* in excitatory neurons through the Seurat AddModuleScore function. Our findings underscored a significant reduction in the average expression of these *SOD1*-correlated cuproptosis genes in ALS across all excitatory neuron subtypes (Fig. 1G). To glean insights into the potential biological ramifications of these findings, we subsequently curated a list of genes that exhibited a positive correlation with both *SOD1* and *FDX1* (Pearson correlation, |*R*| > 0.5, adjusted *P* < 0.05) for subsequent functional GO and KEGG enrichment analyses. Consistently aligned with the known mechanistic underpinnings of cuproptosis and ALS pathogenesis, the most significantly enriched cellular component was the mitochondrion (Fig. 1H), and the associated biological functions encompassed protein folding and mitochondrial respiratory chain complex assembly (Fig. 1I). Furthermore, the KEGG analysis unveiled their involvement in signaling pathways connected with multiple neurodegenerative diseases (Fig. 1J). Collectively, these findings offer evidence to suggest that *SOD1* may contribute to ALS through its close association with genes governing cuproptosis.

In summary, our scrutiny of genetic variants within genes implicated in Cu metabolism and their cell type-specific transcriptomic alterations within the primary motor cortex has unveiled preliminary evidence supporting the intriguing association between cuproptosis genes and ALS. These findings highlighted the yet unclarified roles of Cu in disease pathogenesis, holding the promise of meaningful mechanistic insights and potential therapeutic avenues. Given the dual associations of mitochondrial Cu overload and deficiency with aggregation-induced cell death, future research endeavors may focus on elucidating the specific cellular states that modulate Cu-induced cytotoxicity, leveraging animal models and induced pluripotent stem cell (iPSC)-derived motor neurons from ALS patients to further advance our understanding of cuproptosis in ALS.

## Ethics declaration

Ethical approval for this study was obtained from the PUMCH Research Ethical Boards (No. JS-2624). All participants have provided written consent or permitted a relative to sign on their behalf.

## Author contributions

Study conception & design, DH; performed data analysis, DH and XW; clinical data collection, LC, ML, XY, and DS; patient sample management, CW; Supervision, LC; manuscript drafting, DH. All authors contributed to data interpretation and manuscript revision. All authors read and approved the publication of the final manuscript.

## Conflict of interests

No conflict of interests was disclosed. The funding party is not involved in any aspect pertinent to the study, and none of the authors is financially related to a pharmaceutical company or other agency.

## Funding

This work was supported by the Strategic Priority Research Program (Pilot study) “Biological basis of aging and therapeutic strategies" of the 10.13039/501100002367Chinese Academy of Sciences (No. XDB39040000), National High Level Hospital Clinical Research Funding (China) (No. 2022-PUMCH-B-017), CAMS Innovation Fund for Medical Sciences (China) (2021-I2M-1-003, 2019-I2M-5-066), Shanghai Municipal Science and Technology Major Project (China) (No. 2017SHZDZX01), and 10.13039/501100001809National Natural Science Foundation of China (No. 32288101, 32200536).

## Data availability

The summary of genetic alterations in ALS patients analyzed during this study is included in the supplementary files and deposited in the Genome Variation Map (GVM) in the National Genomics Data Center, Beijing Institute of Genomics, Chinese Academy of Sciences, and China National Center for Bioinformation, under accession number GVM000588. Individual-level sequencing data are available from the corresponding authors on reasonable request.
