# Supplementary material for: Mutational and transcriptional profiling of cuproptosis-associated genes in amyotrophic lateral sclerosis
Source: Genes Dis. 2024 Jan 14;12(1):101208. doi: 10.1016/j.gendis.2024.101208 (PMC11416654; doi:10.1016/j.gendis.2024.101208)
Supplement: Multimedia component 2 [file mmc2.docx]

**Extended Materials**

**Study population**

The cohort includes 508 patients of Chinese descent diagnosed as ALS according to El Escorial criteria, who were recruited between December 2017 and July 2021 at Peking Union Medical College Hospital (PUMCH) (**Table S5**). Motor assessment including ALS Functional Rating Scale–Revised (ALSFRS-R) was performed by experienced neurologists (LC, ML, XY), and the rate of disease progression was defined as changes of ALSFRS-R per month between symptom onset and the first assessment. Blood samples were collected from the patients with written informed consent, which was approved by the PUMCH Research Ethical Boards (No. JS-2624). The patients were followed until January 2023 (DS, CW), with a median follow-up period of 22 months. The sequencing dataset of the HuaBiao Project which involves 4,961 healthy subjects of Chinese ethnicity, was used as control cohort.

**Whole-exome sequencing**

Detailed WES methodology was as described in previous study [1]. Briefly, DNA extracted from the peripheral blood with DNA Isolation Kit (Blood DNA Kit V2, #CW2553) was sheared by Bioruptor UCD-200 (Diagenode). Library was prepared using KAPA Library Preparation Kit (Kapa Biosystems, #KR0453) and SureSelect XT2 Target Enrichment System (Agilent), which was sequenced on the Illumina NovaSeq platform as paired-end 150-bp reads according to Illumina specifications. Illumina Sequence Control Software was used for variant quality control and downstream analysis, with high quality reads aligned to the UCSC human reference genome sequence (build 37.1 version hg19) using Burrows-Wheeler Alignment tool. Variants were called based on GATK Best Practices and annotated using ANNOVAR (version: 2016-05-1110:54:48-0700).

**Variant filtering and interpretation**

To identify genes harboring imbalanced rare variants in ALS cases and to estimate the cumulative effect of rare variants within each gene, the gene-based sequence kernel association test (SKAT) analysis was performed using the HuaBiao cohort as controls (Bonferroni-corrected). The variants that met the following criteria were selected for further evaluation: (1) non-synonymous exonic variants annotated as missense, start-lost, stop-gained, stop-loss or frameshift mutations; (2) minor allele frequency (MAF) lower than 0.001 in Genome Aggregation Database (gnomAD) East Asian population and HuaBiao cohort; (3) significant allelic association with ALS by standard Fisher’s exact test. The in-silico ensemble prediction tools such as M-CAP, CADD and REVEL were used to evaluate the pathogenic potential of these qualifying rare variants. The ALS variants defined as disease-causing or likely disease-causing mutations (DM/DM?) according to Human Gene Mutation Database (HGMD) were screened for all ALS patients.

**Single cell sequencing dataset and data processing**

We obtained the single cell gene expression matrix of human primary motor cortex from GSE174332, which was derived from 17 sporadic ALS patients and 17 pathologically normal controls [2]. The R package Seurat (v4.2.1) 21 was used for normalization, integration, dimensional reduction, clustering, and visualization. Specifically, cells with unique feature counts less than 50 and mitochondria genes greater than 7% were removed from subsequent analyses. The top 2000 most variable genes were selected for downstream PCA dimensional reduction, and the first 15 PCs were used for k-means clustering at a resolution of 0.5. The clustered cells were visualized by Uniform Approximation and Projection method (UMAP), and biologically annotated based on canonical marker genes for neuronal and non-neuronal cells. Subtypes of excitatory and inhibitory neurons were further annotated according to the curation provided in the original manuscript. The expression correlation between SOD1 and cuproptosis genes was calculated and visualized using ggcorrplot package in R. Pearson correlation analysis was performed to select genes correlated to the cuproptosis genes of interest (|R| > 0.5, adjusted p < 0.05), and GO and KEGG enrichment analysis were conducted to functionally characterize the genes, with the top enriched terms displayed in ascending order of p-value (p < 0.05).

**Statistical analysis**

Patients were stratified according to the identified mutations. Age of onset and progression rate was compared with Mann-Whitney test. Kaplan-Meier curves showing survival from disease onset to death, tracheostomy, or censoring were compared with log–rank (Mantel-Cox) test. Cox proportional hazards regression was used to calculate hazard ratio (HR) and 95% confidence interval (CI). P < 0.05 was considered statistically significant. R (version 4.2.2) and GraphPad (version 8.5.1) was used for data processing and statistical analyses.

1. He D, Shang L, Liu Q, et al (2021) Association of apolipoprotein E ε4 allele and amyotrophic lateral sclerosis in Chinese population. Amyotroph Lateral Scler Front Degener. https://doi.org/10.1080/21678421.2021.1953077

2. Pineda, S. Sebastian, Hyeseung Lee, Brent E. Fitzwalter, Shahin Mohammadi, Luc J. Pregent, Mahammad E. Gardashli JM et al. (2021) Single-cell profiling of the human primary motor cortex in ALS and FTLD. bioRvix. https://doi.org/10.1101/2021.07.07.451374
